# Apical Positioned Flap with a Combination of Mucograft and Fibro-Gide for Augmenting Keratinized Gingiva with Thin Gingival Phenotype: A Case Report

**DOI:** 10.1055/s-0045-1810015

**Published:** 2025-07-18

**Authors:** Xin-Rui Zhu, Yi Liu

**Affiliations:** 1 Department of Stomatology, Beijing Haidian Hospital, Beijing, People's Republic of China; 2 Department of Periodontics, School of Stomatology, Capital Medical University, Beijing, People's Republic of China

**Keywords:** Mucograft, Fibro-Gide, keratinized gingiva, thin gingival phenotype, apical positioned flap

## Abstract

The width and thickness of keratinized gingiva play a crucial role in the periodontal health and long-term stability of natural teeth. Noncrosslinked xenogeneic collagen matrix (Mucograft) has demonstrated favorable outcomes in augmenting keratinized gingival width (KGW), though its effect on increasing gingival thickness remains suboptimal. In contrast, crosslinked xenogeneic collagen matrix (Fibro-Gide) effectively enhances keratinized gingival thickness (KGT) but is unable to increase KGW. The aim of this study is to investigate the combined transplantation of Mucograft and Fibro-Gide in patients with thin gingival biotypes and insufficient KGW, evaluating whether this approach can simultaneously achieve both widening and thickening of the keratinized gingiva. This study selected a patient with a thin gingival biotype and insufficient KGW. A horizontal incision was made 0.5 mm above the mucogingival junction, and a partial-thickness flap was elevated and repositioned apically. Fibro-Gide was placed at the apical aspect of the surgical site, enveloped by the partial-thickness flap, while its coronal aspect was positioned in close contact with Mucograft, which was exposed in the surgical area. Both graft materials were securely sutured to the periosteum, ensuring partial contact with the keratinized gingiva. At the 12-month follow-up, the KGW increased from 1.5 to 3.5 mm and the KGT increased from 0.8 to 2.1 mm. For patients with a thin gingival biotype, the combined transplantation of Mucograft and Fibro-Gide can simultaneously augment the width of keratinized gingiva and increase gingival thickness.

## Introduction

The width and thickness of keratinized gingiva are of significant importance for the health of natural teeth and peri-implant tissues. Adequate keratinized gingiva can reduce plaque accumulation and lower the risk of periodontal disease. 1 2 Currently, autogenous tissue, such as free gingival graft and connective tissue graft, is considered the gold standard for patients with insufficient keratinized gingival width (KGW) and keratinized gingival thickness (KGT). However, harvesting autogenous tissue requires a second surgical site, which significantly increases operative time, patient discomfort, and risks such as swelling and bleeding at the donor site. Consequently, there is an urgent clinical need to identify alternatives to autogenous tissue. 3 Mucograft (Geistlich Pharma AG, Wolhusen, Switzerland), a porcine-derived, noncrosslinked, resorbable collagen matrix, has been demonstrated in recent years to achieve KGW augmentation comparable to that of autogenous tissue. 4 5 However, unlike autogenous tissue, which can simultaneously increase both the width and thickness of keratinized gingiva, Mucograft exhibits limited efficacy in augmenting gingival thickness. 6

In recent years, researchers have reported on a volume-stable collagen matrix, Fibro-Gide (Geistlich Pharma AG), which has demonstrated effects comparable to autogenous tissue in augmenting KGT. 7 8 However, as Fibro-Gide cannot be used to increase KGW, patients with deficiencies in both width and thickness of keratinized gingiva typically require two separate surgical procedures for improvement. The present case report explores the combined transplantation of Mucograft and Fibro-Gide to simultaneously enhance both KGW and KGT. This approach offers a potential new strategy for the treatment of thin gingival biotypes with insufficient KGW.

## Case Report

### Case Presentation

**Fig. 1:**
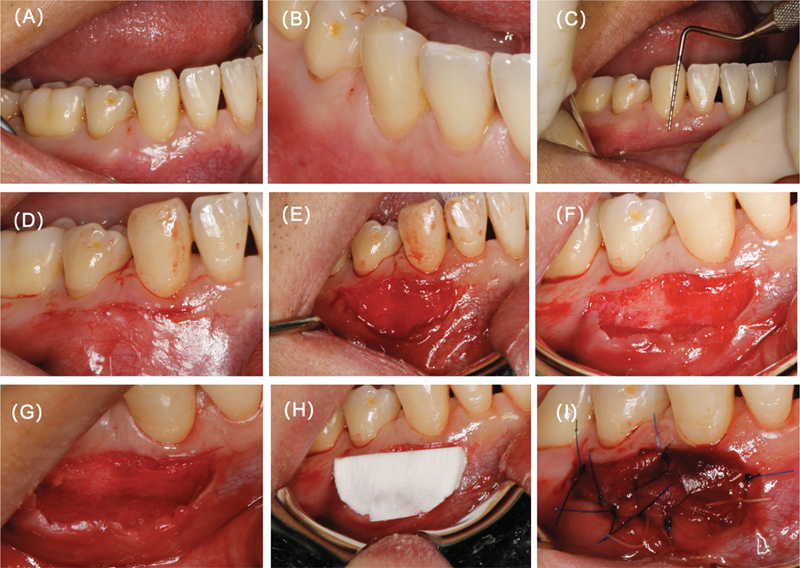
Preoperative findings and surgical procedure. ( **A** ) Buccal aspect showing keratinized mucosa width (KMW) of the canine. ( **B** ) Occlusal aspect showing KMW of the canine. ( **C** ) Periodontal probe showing inadequate KMW of the canine. ( **D** ) Horizon incision made 0.5 mm above the mucogingival junction (MGJ). ( **E** ) Apically positioned flap. ( **F** ) Buccal aspect of Fibro-Gide at the bottom of the flap. ( **G** ) Occlusal aspect of Fibro-Fide at the bottom of the flap. ( **H** ) Mucograft placed on the periosteum. ( **I** ) Stabilization of Mucograft and Fibro-Gide.

A 32-year-old man with no systemic conditions or diseases presented to the hospital on March 10, 2024. During routine periodontal follow-up 1 year after completing orthodontic treatment, progressive gingival recession was observed in teeth 43 and 44. Mild gingival recession was noted in teeth 43 and 44 ( Fig. 1A ), with a relatively thin keratinized gingiva (KGT ≤ 1 mm with endodontic file; Fig. 1B ). The buccal KGW of teeth 43 and 44 measured 1.5 mm with periodontal probe, indicating insufficient buccal KGW ( Fig. 1C ). The patient expressed a desire to prevent further gingival recession and achieve long-term stability. The patient provided and signed an appropriate informed consent statement.

### Case Management

**Fig. 2:**
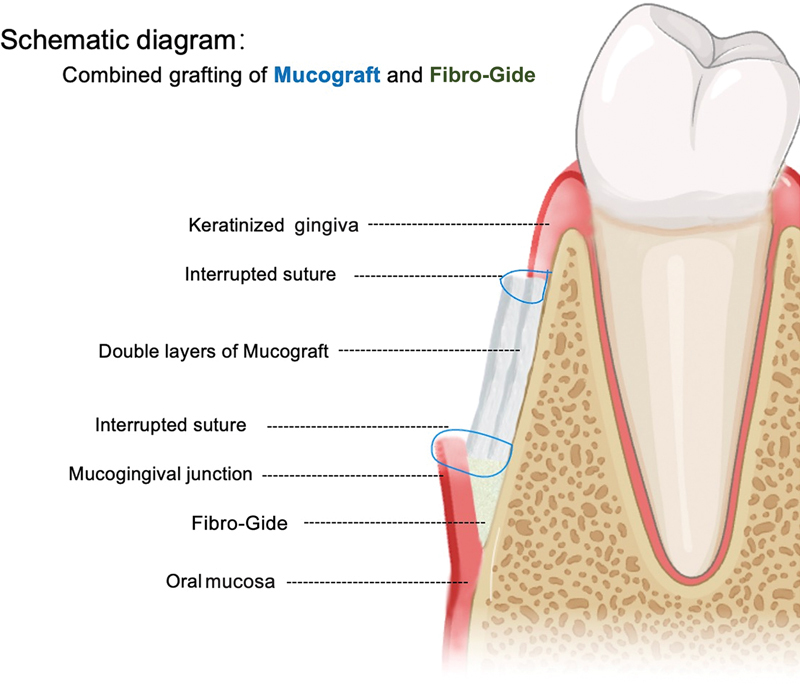
Schematic diagram of the surgical method.

Following a full-mouth examination, the patient underwent supragingival scaling and received oral hygiene instructions (OHI). Local anesthesia was administered using 1:100,000 epinephrine (Produits Dentaires Pierre Rolland, Acteon Pharma Division, Merignac, France). The flap design utilized a root-oriented flap technique. A horizontal incision was made 0.5 mm above the mucogingival junction using a no. 15C blade, extending from the mesial aspect of tooth 43 to the distal aspect of tooth 44 ( Fig. 1D ). A split-thickness flap was carefully elevated and repositioned apically 8 mm ( Fig. 1E ). Fibro-Gide was trimmed in the crown–root direction to 3 mm, which was placed apical to the recipient site, with its buccal and apical surfaces enveloped by the split-thickness flap ( Fig. 1F, 1G ). The coronal aspect of Fibro-Gide was closely approximated to a double-layered Mucograft crown–root direction trimming to 5 mm, which positioned within the surgical site. Both Fibro-Gide and Mucograft were securely sutured to the periosteum, keratinized gingiva, and split-thickness flap using interrupted sutures, ensuring contact between the local keratinized gingiva at the apical portion of the surgical site and both graft materials. Mucograft was left exposed for open healing ( Fig. 1H ). The coronal margin was fixed to the keratinized gingiva with interrupted sutures to prevent displacement of the graft materials during tissue healing ( Fig. 1I ). The surgical approach diagram can more intuitively show the placement of the material and its anatomical relationship with the surrounding tissues ( Fig. 2 ).

The patient was instructed to apply ice packs to the surgical site within the first 6 hours postsurgery. They were prescribed 250 mg cefuroxime axetil every 12 hours for 6 days and 400 mg loxoprofen for pain relief as needed. For the first 2 weeks, patients were advised to avoid brushing the surgical site and consuming hard foods to prevent mechanical injury, instead consuming soft foods, brushing nonsurgical areas twice daily with a soft-bristled toothbrush, and rinsing with 0.12% chlorhexidine mouthwash three times daily for 1 minute each time. Sutures were routinely removed after 14 days.

The width and thickness of the keratinized gingiva were collected before and 12 months after surgery. The measurement method is as follows:

- *KGW:* The distance from the midpoint of the buccal gingival margin of teeth 44 and 43 to the gingival junction was measured using a periodontal probe.
- *KGT:* This is assessed by inserting an endodontic file with a rubber stopper into the midpoint of the line connecting the midpoint of the buccal gingival margin of teeth 44 and 43 to the gingival junction until the tip contacted the bone surface. After positioning the rubber stopper against the gingiva, the file was removed, and the distance from the tip to the stopper was measured using a digital caliper with 0.1-mm precision. 9

## Results

**Fig. 3:**
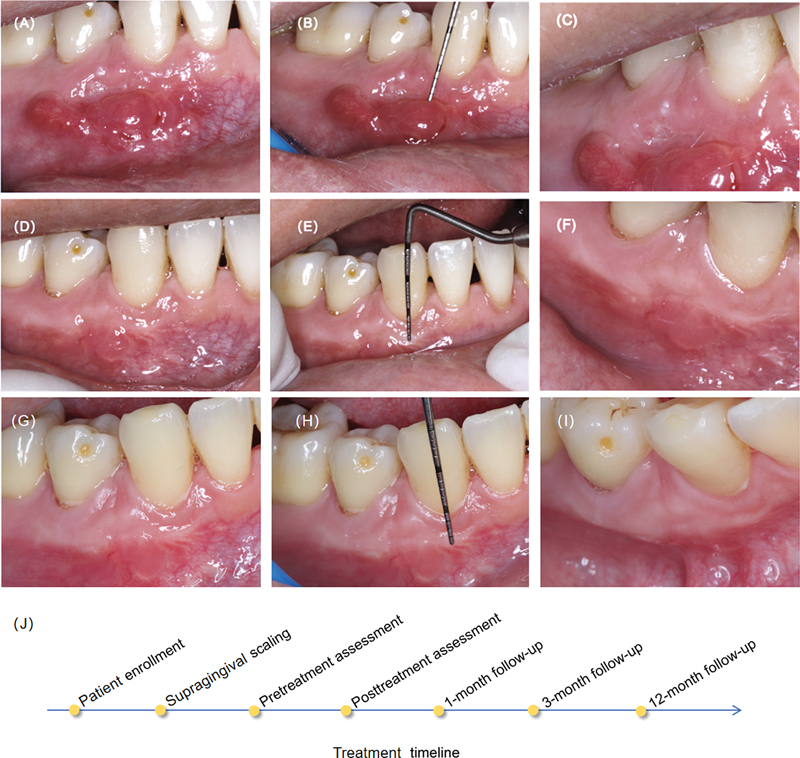
Follow-up findings. ( **A** ) Keratinized gingival width (KGW) on the buccal aspect at 1 month. ( **B** ) Measurement of KGW on the buccal aspect at 1 month. ( **C** ) Healing of buccal keratinized gingiva at 1 month. ( **D** ) KGW on the buccal aspect at 3 months. ( **E** ) Measurement of KGW on the buccal aspect at 3 months. ( **F** ) Formation of buccal keratinized gingival thickness (KGT) at 3 months. ( **G** ) KGW on the buccal aspect at 12 months. ( **H** ) Measurement of KGW on the buccal aspect at 12 months. ( **I** ) Formation of buccal KGT at 12 months. ( **J** ) Timeline for the follow-up.

All sutures were removed 14 days postsurgery. At 1 month, an increase in KGW was observed ( Figs. 3A , 3B ), whereas no significant improvement in KGT was noted ( Fig. 3C ). By 3 months, the newly gained KGW remained stable ( Fig. 3D , 3E ), and a significant increase in KGT was evident ( Fig. 3F ). By 12 months, the newly gained KGW and thickness remained stable ( Fig. 3G–I ). At the 12-month follow-up, the KGW of teeth 43 and 44 had both increased from a preoperative measurement of 1.5 to 3.5 mm, and the KGT of teeth 43 and 44 had both risen from a preoperative value of 0.8 to 2.1 mm. The surgical sites where Mucograft and Fibro-Gide were implanted both exhibited favorable thickening outcomes. No adverse and unanticipated events occurred. The patients were satisfied with the results achieved and reported that the treatment process and surgical reaction were acceptable. The visual analog scale (VAS) score for postoperative pain was 2.

## Discussion

This study investigated the combined transplantation of Mucograft and Fibro-Gide in a thin gingival phenotype patient with insufficient KGW. The results demonstrated that, at 12 months postsurgery, the width of keratinized gingiva increased by 2 mm, and the thickness of keratinized gingiva increased by 1 mm. These outcomes are consistent with the effects reported in previous studies where the two materials were used individually, indicating that the combined transplantation of Mucograft and Fibro-Gide can simultaneously widen and thicken the keratinized gingiva without compromising the clinical efficacy of either material alone. 10 11 12 13 14 15 Previous research showed that a KGW of more than 2 mm and a thickness of more than 1 mm are conducive to maintaining periodontal health, which indicated a well-supported periodontal health and long-term stability. 16 17 18

Mucograft and Fibro-Gide are both porcine absorbable collagen matrices. This homology is conducive to enhancing the effect of combined transplantation. 19 20 The difference is that Mucograft has a loose structure and a fast degradation rate, which are suitable for epithelial cells to widen keratinized tissue and can be exposed for healing. Fibro-Gide has enhanced mechanical properties and biodegradability, which can effectively maintain the tissue stability of the defect area, thereby promoting the formation of thicker connective tissue and requiring buried healing. 21 Considering that both materials require sufficient blood supply and cell sources to induce keratinized gingiva formation, we arranged the materials side by side on the periosteum. Fibro-Gide was placed on the radicular side of the surgical area, and the semi-thick flap was covered and tightly combined with the coronal Mucograft. 22 23 It is worth noting that although the two materials were not stacked, according to the KGT measurement method selected in this study and the material trimming size, the KGT of the Mucograft implant area showed an increasing trend during the 12-month follow-up period. This finding contradicts the view of previous studies that Mucograft is not effective in thickening keratinized gingiva, suggesting that the combined transplantation of Mucograft and Fibro-Gide has a synergistic effect in improving keratinized gingiva. 24 This may be due to the different degradation cycles of the two materials. The volume-stable Fibro-Gide located at the root can provide thickening space for the widened keratinized gingiva for a long period of time after the rapid degradation of Mucograft, forming a “wedge effect.”

The significant shrinkage rate following xenogeneic collagen matrix (XCM) transplantation remains a critical challenge in the clinical application. Previous studies have reported shrinkage rates ranging from 40 to 62% for Mucograft 25 and from 33 to 85% for Fibro-Gide. 15 In the present case, the shrinkage rates were 65% for Mucograft and 75% for Fibro-Gide, aligning with prior research but indicating relatively high values. This outcome may be associated with insufficient localized blood supply in a patient with thin gingival phenotype, as well as following the combined transplantation of the two XCMs. 2 Studies have explored the simultaneous use of platelet-rich fibrin (PRF) with XCM to widen keratinized gingiva, demonstrating that PRF reduces the early shrinkage rate of XCM and enhances the widening effect. 26 Therefore, for patients with a thin gingival phenotype, the concurrent application of plasma-derived products alongside the combined transplantation of Mucograft and Fibro-Gide could be considered to reduce shrinkage rates and further improve the precision of the further treatment.

This study has certain limitations. First, the current report only conducted a 12-month follow-up observation of this novel surgical approach, and its long-term efficacy and patient benefits require further investigation. However, previous literature has reported that the primary changes of XCM occur within the first 6 months. 27 Moreover, based on the existing results, the combined transplantation of the two materials demonstrates the potential to effectively address the shortcomings of single-material transplantation, thereby reducing the need for multiple surgeries and minimizing associated patient harm. Subsequent basic research is needed to further clarify the effect of the combined application of the two materials. Additionally, future studies should aim to increase sample size and design case-control studies to further validate the effectiveness of this surgical technique.

## Conclusion

For patients with a thin gingival phenotype, the combined transplantation of Mucograft and Fibro-Gide can simultaneously widen the keratinized gingiva and thicken the gingival tissue, achieving outcomes comparable to those obtained with stand-alone transplantation.
